# Armed assault of Russia on Ukraine and looming danger on regional mental health: a call for action by psychiatrists in conflict settings

**DOI:** 10.1017/gmh.2022.32

**Published:** 2022-10-03

**Authors:** Sheikh Shoib, Aishatu Yusha'u Armiya'u, Miyuru Chandradasa, Albina Zharkova, Pavlo Kolesnyk, Sarya Swed, Don Eliseo Lucero-Prisno

**Affiliations:** 1 Department of Psychiatry, Jawahar Lal Nehru Memorial Hospital, Srinagar, Kashmir, India; 2 Department of Psychiatry, Forensic Unit, Jos University Teaching Hospital, Jos, Plateau state, Nigeria; 3 Department of Psychiatry, University of Kelaniya, Ragama, Sri Lanka; 4 Department of Family Medicine, Sumy State University, Sumy, Ukraine; 5 Department of Family Medicine and Outpatient Care, Uzhhorod, Ukraine; 6 Faculty of Medicine, Aleppo University, Aleppo, Syrian Arab Republic; 7 Department of Global Health and Development, London School of Hygiene and Tropical Medicine, London, UK

The Russian-Ukrainian socio-political relationship is embroiled in centuries of conflicts, bloodshed and brutal invasions. The modern dissension dates back to the 2014 annexation of Crimea by Russia in the aftermath of the Ukrainian Revolution of Dignity and has been escalating rapidly during the recent months. The tensions between the two nations have boiled over to the Russian forces entering Ukrainian soil from the North, South, and East on 24 February 2022. This puts the lives of millions of people at risk, particularly those residing in Eastern Ukraine who are estimated to be around 14 million people and also residents of major cities in other parts of the country (Ukraine, 2022). The ongoing armed conflict will have a deleterious impact on their mental health and social wellbeing. The fear of further armed aggression will force people to leave their homes and will lead to dire mental health consequences (Jazeera, 2022).

Armed interventions will have a disastrous impact on the mental health of individuals in conflict-affected regions. According to new WHO estimates, the prevalence of mental health disorders in conflict settings at any point in time is estimated to be 22.1% (Charlson *et al*., 2019). Applying these estimates to the populations in the conflict-affected areas in Ukraine suggests that millions of people are at risk of negative psychiatric consequences, with many potentially requiring urgent psychological support.

More than 3000 civilians have perished and over 7000 have been injured as a result of the fighting in Eastern Ukraine between 2014 and September 2021. As of mid-December 2021, 1.46 million individuals had been displaced. As of now the Russian troops have entered Ukraine and within the first day of the clashes more than 100 lives have been lost and the situation is likely to deteriorate further (News, 2022). Drivers of immediate humanitarian needs in Eastern Ukraine are the ongoing armed conflict, movement restrictions due to imposed martial law, curfew in major cities, fathers separated from families to fight and persisting COVID-19 pandemic (Ukraine-Russia-News, 2022). Further, war escalation in Eastern Ukraine and other cities is projected to result in major casualties and an increase in humanitarian needs.

Already in Eastern Ukraine, 3.4 million people, 60% of whom are women and children, require humanitarian assistance after 8 years of conflict. Tens of thousands of civilians have been killed, displaced from their homes. The situation is particularly detrimental and prolonged for people who have lived in the conflict's hotspots such as Donetsk and Luhansk (reliefweb, 2020). Shelling, landmines and unexploded ordnance in the past few years pose serious dangers to children in these regions. Their lives are jeopardized as a result of the conflict, which has destroyed key civilian infrastructures such as health clinics, schools and water sources. Education, which is so important for a child's sense of ‘normalcy’, has been broken, with more than one out of every five schools in eastern Ukraine being damaged or destroyed. Teachers and psychologists describe nightmares, social isolation and panic attacks prompted by loud noises as symptoms of significant psychological distress in youngsters. In Donetsk and Luhansk, it is estimated that one out of every four children requires psychosocial assistance (UNICEF, 2017; RESPNSES, 2022). Few people, however, receive that assistance since the available services are overburdened.

The ongoing armed excursion has caused physical injury, death, psychological trauma and internal displacement in Ukraine, and disruptions of healthcare services, transport, education and basic amenities have occurred. In this context, we, a group of psychiatrists experienced in mental healthcare in conflict-affected countries, are providing recommendations to protect the psychological well-being of people in Ukraine as summarized in Panel box 1.

Panel box 1. Recommendations to protect the psychological well-being of people in Ukraine

An expert committee to be appointed with mental health experts including psychiatrists, psychologists, general practitioners, social workers and other relevant professionals

The expert committee to comprise of mental health professionals from Ukraine, other European countries and conflict-affected settings in the world

The committee to liaise with intergovernmental organizations, humanitarian agencies, funding bodies and international mental health professional associations

Develop and initiate a mental health support programme for Ukrainian people addressing acute and intermediate measures

The committee to consider and learn from experiences in the Eastern Ukraine conflict and other regional wars

The expert committee to facilitate creating an app in the Ukrainian language that has simplified, short screening tools for psychological distress and trauma

Create social media platforms in Ukrainian and Russian languages to provide written, audio and video material on psychological consequences of stress and psychological first aid

Distribute psychological first aid manuals in local languages for health care providers, teachers or trained volunteers.

Toll-free psychological support hotline in the Ukrainian language, which could be operated from a neighbouring country during the ongoing conflict

Specialists, trained volunteers and supervised community workers to offer and provide online supportive counselling, online group psychotherapy and advice to primary contact physicians

Schools to remain open virtually at least for a few hours of the day and teachers to focus on emotional support and reconciliation

Field workers to screen for psychological trauma in displaced families and provide psychological first aid using written audio and video material
